# Clinical decision support systems for diabetic foot ulcers: a scoping review

**DOI:** 10.1590/1980-220X-REEUSP-2023-0218en

**Published:** 2024-02-09

**Authors:** Thiago Santos Garces, Açucena Leal de Araújo, George Jó Bezerra Sousa, Virna Ribeiro Feitosa Cestari, Raquel Sampaio Florêncio, Samuel Miranda Mattos, Lara Lídia Ventura Damasceno, Jênifa Cavalcante dos Santos Santiago, Vera Lucia Mendes de Paula Pessoa, Maria Lúcia Duarte Pereira, Thereza Maria Magalhães Moreira

**Affiliations:** 1 Universidade Estadual do Ceará, Programa de Pós-Graduação em Saúde Coletiva, Fortaleza, CE, Brazil.; 2 Universidade Estadual do Ceará, Programa de Pós-Graduação em Cuidados Clínicos em Enfermagem e Saúde, Fortaleza, CE, Brazil.; 3 Secretaria da Saúde do Estado do Ceará, Fortaleza, CE, Brazil.; 4 Universidade Federal do Ceará, Departamento de Enfermagem, Fortaleza, CE, Brazil.

**Keywords:** Diabetes Mellitus, Diabetic Foot, Decision Support Systems, Clinical, Review, Diabetes Mellitus, Pie Diabético, Sistemas de Apoyo a Decisiones Clínicas, Revisión, Diabetes Mellitus, Pé Diabético, Sistemas de Apoio a Decisões Clínicas, Revisão

## Abstract

**Objective:**

Map the scientific evidence on the use of clinical decision support systems in diabetic foot care.

**Method:**

A scoping review based on the JBI Manual for Evidence Synthesis and registered on the Open Science Framework platform. Searches were carried out in primary and secondary sources on prototypes and computerized tools aimed at assisting patients with diabetic foot or at risk of having it, published in any language or period, in eleven databases and grey literature.

**Results:**

A total of 710 studies were identified and, following the eligibility criteria, 23 were selected, which portrayed the use of decision support systems in diabetic foot screening, predicting the risk of ulcers and amputations, classifying the stage of severity, deciding on the treatment plan, and evaluating the effectiveness of interventions, by processing data relating to clinical and sociodemographic information.

**Conclusion:**

Expert systems stand out for their satisfactory results, with high precision and sensitivity when it comes to guiding and qualifying the decision-making process in diabetic foot prevention and care.

## RESUMO

**Objetivo:**

Mapear as evidências científicas sobre uso de Sistemas de Apoio à Decisão Clínica no pé diabético.

**Método:**

Revisão de escopo fundamentada no JBI *Manual for Evidence Synthesis* e registrada na plataforma *Open Science Framework*. Realizaram-se buscas, em fontes primárias e secundárias, sobre protótipos e ferramentas informatizadas direcionadas à assistência ao paciente com pé diabético ou em risco de tê-lo, publicados em qualquer idioma ou período, em onze bases de dados e literatura cinza.

**Resultados:**

Foram identificados 710 estudos e, após critérios de elegibilidade, foram selecionados 23 que retratam o uso de sistemas de apoio à decisão no rastreio do pé diabético, predição do risco de úlceras e amputações, classificação do estágio de gravidade, decisão quanto ao plano de tratamento e avaliação da efetividade das intervenções, por meio do processamento de dados referentes a informações clínicas e sociodemográficas.

**Conclusão:**

Os sistemas especialistas destacam-se por resultados satisfatórios, com alta precisão e sensibilidade no que tange à orientação e qualificação do processo de tomada de decisão na prevenção e no cuidado ao pé diabético.

## RESUMEN

**Objetivo:**

Mapeo de la evidencia científica sobre el uso de sistemas de apoyo a la toma de decisiones clínicas en el cuidado del pie diabético.

**Método:**

Revisión de alcance basada en el Manual de Síntesis de la Evidencia del JBI y registrada en la plataforma Open Science Framework. Se realizaron búsquedas en fuentes primarias y secundarias sobre prototipos y herramientas informatizadas dirigidas a la asistencia de pacientes con pie diabético o en riesgo de padecerlo, publicadas en cualquier idioma o periodo, en once bases de datos y literatura gris.

**Resultados:**

Se identificaron 710 estudios y, tras cumplir los criterios de elegibilidad, se seleccionaron 23, que retrataban el uso de sistemas de apoyo a la toma de decisiones en el diagnóstico del pie diabético, la predicción del riesgo de úlceras y amputaciones, la clasificación del estadio de gravedad, la decisión sobre el plan de tratamiento y la evaluación de la eficacia de las intervenciones, mediante el procesamiento de datos relativos a la información clínica y sociodemográfica.

**Conclusión:**

Los sistemas expertos destacan por sus resultados satisfactorios, con gran precisión y sensibilidad a la hora de orientar y cualificar el proceso de toma de decisiones en la prevención y el cuidado del pie diabético.

## INTRODUCTION

Diabetic foot ulcers are a late complication of diabetes mellitus (DM) and are significantly associated with morbidity, hospitalization and mortality ^(1)^. It is estimated that the lifetime incidence of developing this complication is between 19% and 34%, and recurrence rates vary between 40% within one year of healing and 65% within five years^(2)^.

The event in question is costly to health systems worldwide, given its association with outcomes such as amputation, death, and loss of years of productivity, as well as the complexity of the treatment^(3)^. In Brazil, it is estimated that the annual medical costs of diabetic foot disease are R$586.1 million, 87% of which is spent on outpatient care and 13% on hospitalization. Of this amount, R$498.4 million is spent on treating patients with ulcerated neuro-ischemic feet^(4)^.

Faced with this problem, clinical guidelines and DM manuals recommend screening for the risk of diabetic foot ulceration, as well as providing flows and protocols for managing the complication. However, they vary substantially in terms of the evidence and variables used to support the recommendations, culminating in great variation in clinical signs, tests, and implementation of interventions in an intuitive way and without standardization^(5)^.

Decision support systems (DSS), also known as expert systems, are a valuable tool for qualifying the evidence base in clinical decision-making, as they enable the integration of different techniques and approaches to information management, providing simplified risk assessment and recommendation of specific interventions with high prognostic value, based on individual characteristics and scientific evidence^(6,7)^.

Based on the evidence presented, DSS are useful for predicting diabetic foot risk, as well as for guiding, qualifying, and standardizing decision-making, with a view to preventing outcomes such as amputations and death^(8,9)^. In a preliminary search, a systematic review identified clinical decision support models aimed at different types of chronic wounds, while the only study that addressed a support model for the management of diabetic foot lesions used resources proposed by specialists and based on clinical studies^(10)^.

However, no mapping reviews were found that specifically address the use of DSS in the treatment of diabetic foot ulcers or people at imminent risk of ulceration. This gap motivates the investigation and updating of knowledge on the subject, with the aim of providing support for health professionals, researchers, and decision-makers. In light of this, the present study aims to map the evidence on the use of clinical decision support systems in diabetic foot care.

## METHOD

### Study Design

This is a scoping review, based on the JBI Manual for Evidence Synthesis^(11)^ and the reporting recommendations of the Preferred Reporting Items for Systematic reviews and Meta-Analyses extension for Scoping Reviews (PRISMA-ScR) checklist^(12)^. The final protocol was registered on the Open Science Framework (OSF) platform on May 17, 2022, with DOI identification 10.17605/OSF.IO/UWTH6.

The study was conducted in five stages: 1) identification of the research question; 2) survey of relevant studies; 3) selection of studies according to pre-established criteria; 4) categorization of data; and 5) presentation of results.

### Research Questions

The Population, Concept, and Context (PCC) strategy was used to construct the research question. The participants (P) in this review were people diagnosed with DM, the concept (C) addressed was the clinical decision support system, and the context (C) was people with diabetic foot or imminent risk. Thus, the following main guiding question was formulated:

– What is the scientific evidence on the use of DSS in the health care of people with diabetic feet or at risk of having them?

Secondary questions were:

– What do DSS for diabetic foot generally address?

– How can DSS for diabetic foot be categorized?

– What are the main advances and gaps in research on DSS for diabetic foot?

### Eligibility Criteria

Studies that presented some prototype or functional computerized tool, applicable or applied to the care of patients at risk or with diabetic foot, published in any language or period, were included. As for the type of study, observational and experimental, quantitative and/or qualitative studies with primary or secondary data were chosen. Letters to the editor, abstracts, and studies in the design phase were excluded, as they do not provide concise results on DSS in clinical practice.

### Search Strategy

The search strategy was built using the Health Sciences Descriptors (DeCS) and Medical Subject Headings (MeSH) databases, together with Boolean operators AND and OR, as shown in Chart 1 ^(13)^.

**Chart 1:** Search strategy – Fortaleza, CE, Brazil, 2022.

	P	C	C
**Extraction**	People with Diabetes Mellitus	Decision Support Systems	Diabetic Foot
**Conversion**	Diabetes Mellitus	Decision Support Systems	Diabetic Foot
**Combination**	Diabetes Mellitus; Diabetes; Diabetic; Diabetic Patient	Decision Support Systems; Clinical Decision Support Systems; Clinical Decision Support; Specialist Systems	Diabetic Foot; Diabetic Feet; Diabetic Foot Ulcer
**Construction**	“Diabetes Mellitus” OR Diabetes OR Diabetic OR “Diabetic Patient”	“Decision Support Systems” OR “Clinical Decision Support Systems” OR “Clinical Decision Support” OR “Specialist Systems”	“Diabetic Foot” OR “Diabetic Feet” OR “Diabetic Foot Ulcer”
**Use**	(“Diabetes Mellitus” OR Diabetes OR Diabetic OR “Diabetic Patient”) AND (“Decision Support Systems” OR “Clinical Decision Support Systems” OR “Clinical Decision Support” OR “Specialist Systems”) AND (“Diabetic Foot” OR “Diabetic Feet” OR “Diabetic Foot Ulcer”)		

The search took place on October 23, 2022, in the following databases: Scientific Electronic Library Online (SciELO), Medical Literature Analysis and Retrieval System Online (MEDLINE), PubMed, Scopus, Web of Science, ScienceDirect, Cumulative Index to Nursing and Allied Health Literature (CINAHL), Latin American and Caribbean Health Sciences Literature (LILACS), Cochrane Library, and Embase. Gray literature was retrieved from the Brazilian Digital Library of Theses and Dissertations (BDTD), the Catalogue of Theses and Dissertations (CTD) of the Coordination for the Improvement of Higher Education Personnel (CAPES), and the Open Access Theses and Dissertations (OATD).

### Selection of Studies

The results obtained from the databases were imported into the Rayyan®^(14)^, reference manager, developed by the Qatar Computing Research Institute (QCRI). At this point, duplicates were removed, and the studies were independently selected and screened by two researchers, with any discrepancies being resolved with the participation of a third examiner with experience in the field.

After removing the duplicates, the articles were selected by reading their titles and abstracts based on the pre-established study criteria. The studies included in the first stage were then read in their entirety to check for permanence. Justification was given for the articles that were excluded. All the references of the included articles were checked for other potentially relevant studies. Finally, the identification and selection stages were documented using the Preferred Reporting Items for Systematic Reviews and Meta-Analyses flowchart^(12)^.

### Mapping and Data Analysis

A data extraction strategy was defined and adapted according to the JBI manual in order to select the following relevant information: 1) characterization: author, country, journal, theme, year, title, objectives, and type of study; 2) clinical applicability; 3) type of technology used; and 4) main results and limitations, which were organized in the form of tables with narrative content in Microsoft Excel®.

## RESULTS

**Figure 1:**
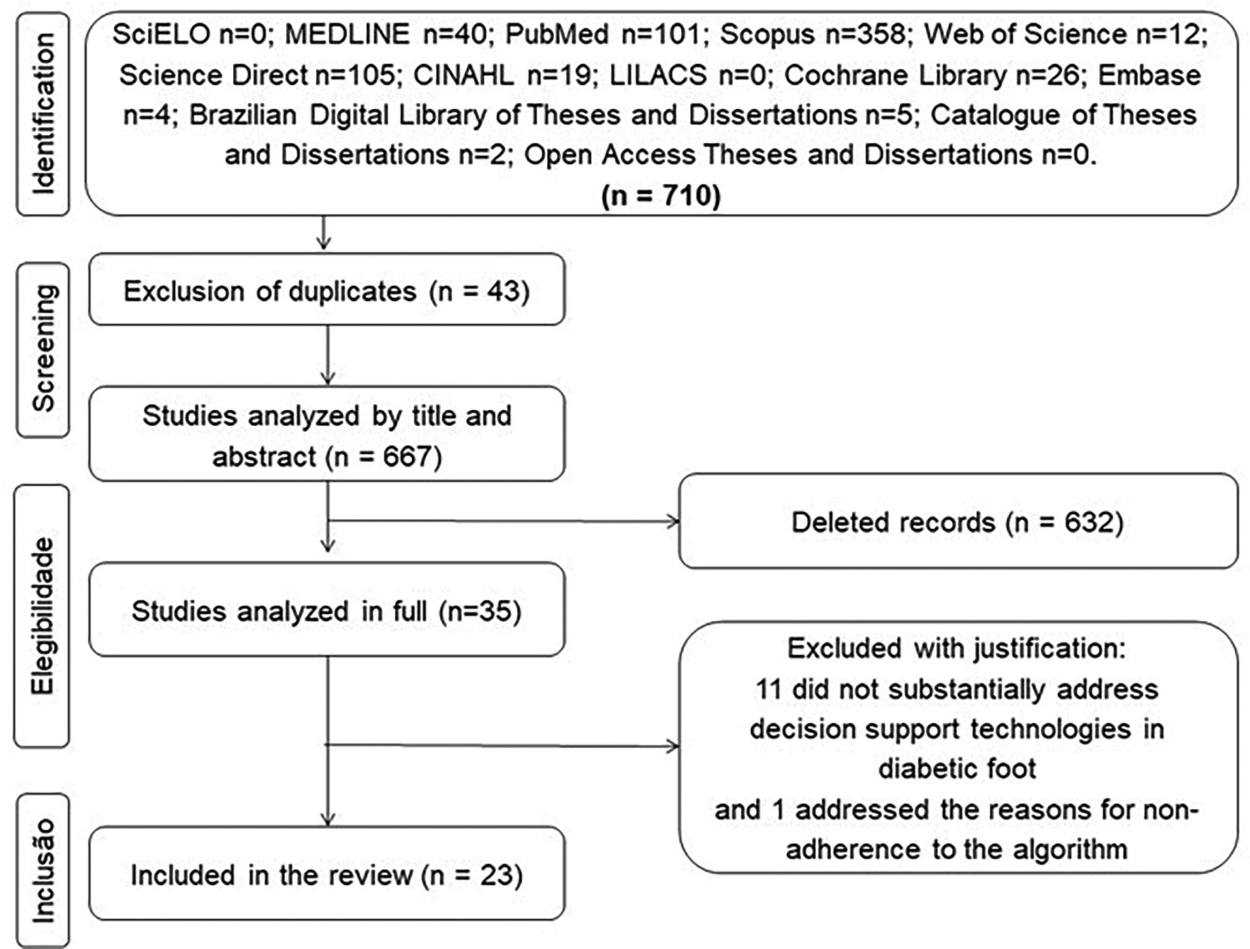
Search flowchart, according to the PRISMA-ScR recommendations – Fortaleza, CE, Brazil, 2022.

The search in the information sources resulted in 710 studies, of which 43 were excluded because they were duplicates, leaving 667 publications. Titles and abstracts were analyzed, and 632 were excluded by applying the eligibility criteria. As a result, 35 studies were fully analyzed and, of these, 23 met the primary and secondary questions of the study, as shown in Figure 1.

Chart 2 shows the characterization and summary of the articles mapped and included in this review. In terms of place of origin, the majority of the studies were carried out on the European continent (n = 10; 43.4%), followed by the Asian continent (n = 8; 34.7%), with three studies (n = 3; 13.0%) from the American continent, and two (n = 2; 8.6%) from Oceania.

**Chart 2:** Characterization and synthesis of the articles mapped – Fortaleza, CE, Brazil, 2022.

No.	Author/Year/Country/Journal	Objectives	Technology used	Clinical applicability	Main results
1.	Bender C, Cichosz SL, Malovini A, Bellazzi R, Pape-Haugaard L, Hejlesen O (2021)/Denmark/Journal of Diabetes Science and Technology^(9)^	Build a prototype of an interactive teaching tool, using case-based reasoning, for evidence-based diabetic foot ulcer care.	Machine learning/Case-based reasoning^(9)^	Educational tool for nurses for diabetic foot care and screening.	The prototype is capable of calculating a dissimilarity score that provides a quantitative measure between a new case and stored cases.
2.	Casal-Guisande M, Cerqueiro-Pequeño J, Comesaña-Campos A, Bouza-Rodríguez JB (2020)/Spain/Diabetic Medicine^(7)^	Adapting a methodology based on expert systems to monitor patients prone to developing diabetic foot wounds.	Machine learning/Decision manager supported by fuzzy inference^(7)^	Determining the risk of developing diabetic foot and evaluating the effectiveness of the care received.	The system includes the initial stage of data collection, by taking a photo of the lesion and filling in a questionnaire on risk factors, followed by image processing (Wagner scale), calculation, and modeling of the results for interpretation and generation of alerts, decision-making, and application of treatment.
3.	Chappell FM, Crawford F, Horne M, Leese GP, Martin A, Weller D, et al (2021)/United Kingdom/BMJ open Diabetes Research & Care^(15)^	Developing and validating a clinical prediction rule for foot ulceration in people with diabetes.	Clinical prediction rule^(15)^	Predicting the risk of diabetic foot ulceration through plantar thermal imaging analysis.	The clinical prediction rule scores (0, 1, 2, 3, and 4) show a two-year ulcer risk of 2.4%, 6.0%, 14.0%, 29.2%, and 51.1%, respectively. It is a simple tool that uses routinely obtained data and helps prevent ulcers by directing care to patients with a score of 1 or more.
4.	Crawford F, Bekker HL, Jovem M, Sheikh A (2010)/United Kingdom/Journal of Innovation in Health Informatics^(16)^	Understanding the vision of primary health care professionals in relation to diabetic foot disease screening and their experience with the SCI-DC system.	Machine learning^(16)^	Diabetic foot screening.	SCI-DC is an information system designed to create a shared electronic record for use in the care of patients with DM. There were favorable perspectives on the system, especially with regard to the foot screening screens, the transfer of information from primary to secondary care, the reduction of variability in information from podiatrists, and the source of information for auditing purposes.
5.	Crawford F, Cezard G, Chappell FM (2018)/United Kingdom/Diabetic Medicine^(17)^	Developing and validating a prognostic model of independent risk factors for foot ulceration in diabetes.	Clinical prediction rule^(17)^	Predicting the risk of diabetic foot ulceration through plantar thermal imaging analysis.	A simple prognostic model was developed with three independent predictive risk factors that were statistically associated with diabetic foot ulcers: history of ulceration/inability to feel a 10g monofilament/at least one absent pulse.
6.	Cruz-Vega I, Peregrina-Barreto H, Rangel-Magdaleno JJ, Ramires-Cortes MJ (2019)/New Zealand/IEEE Xplore^(18)^	Comparing intelligent classifiers of diabetic foot thermal patterns in patients with diabetes mellitus and a control group.	Machine learning^(18)^	Predicting the risk of diabetic foot ulceration through plantar thermal imaging analysis.	The results of using support vector machines and multi-layer perception neural networks to classify medical image patterns are highly accurate and satisfactory. However, the use of deep learning is gaining momentum, given the increased accuracy and dispensability of feature extraction and pattern segmentation.
7.	Gamage C, Wijesinghe I, Perera I (2019)/Sri Lanka/IEEE Xplore^(19)^	Using a convolutional neural network to predict stages of diabetic foot severity.	Machine learning/Convolutional neural networks^(19)^	Classification of the stage of diabetic foot severity according to Wagner’s criteria using images.	The dataset of wound images was subdivided for experimentation with pre-trained convolutional neural networks. Among the decision algorithms, artificial neural networks performed most successfully.
8.	Goulionis JE, Vozikis A, Benos VC, Nikolakis D (2010)/Greece/ClinicoEconomics and Outcomes Research^(20)^	Assessing the cost-benefit ratio of two treatments (medical treatment and amputation) in patients with diabetic foot syndrome, using a decision algorithm.	Heuristic decision algorithm, based on the partially observable Markov decision process^(20)^	Evaluation of the cost-effectiveness of medical treatment and diabetic foot amputation.	A simple model for cost-effective decision-making for diabetic foot treatment was created, explaining two pathways between primary clinical data and early and efficient medical decision-making. The use of the model provided improved quality of care, cost-effective clinical decision-making, and adaptability and transferability across different healthcare settings.
9.	Das SK, Roy P, Mishra AK (2022)/India/Concurrency and Computation Practice and Experience^(21)^	Merging high-level resources based on machine learning with low-level and convolutional neural networks to improve the automatic diagnosis of diabetic feet.	Machine learning/Convolutional neural networks^(21)^	Predicting risk and diagnosing diabetic foot ulceration through images.	The fusion of resources from classifiers of different machine learning, logistic regression, support vector machine, and artificial neural networks showed better results in identifying the diabetic foot. Logistic regression outperformed all the evaluation metrics, achieving 95.23% sensitivity and 95.37% specificity.
10.	Deschamps K, Matricali GA, Desmet D, Roosen P, Keijsers N, Nobel F, et al. (2016)/Belgium/Gait & Posture^(22)^	Determine measures of effectiveness of a diabetic foot prediction system based on plantar pressure, analyzing the recognition rate, sensitivity, specificity, as well as its usefulness in implementing pressure distribution strategies.	Semi-automatic total mapping to identify regional metrics^(22)^.	Predicting the risk and diagnosing diabetic foot ulceration through plantar grip measurements.	The comparison of the four groups associated with the classification system highlighted distinct regional differences. The overall recognition rate exceeded 90% for all cross-validation subsets. The sensitivity and specificity of the four groups associated with the classification system exceeded the 0.7 and 0.8 level, respectively.
11.	Farzi S, Kianian S, Rastkhadive I (2018)/Iran/IEEE Xplore^(23)^	Identifying the best classification algorithm to detect diabetes complications.	Machine learning/Decision tree, Random forest, Multi-layer perception neural network, Naive Bayes, Radial base function^(23)^	Predicting risk and diagnosing diabetic foot ulceration through sociodemographic and clinical variables.	The Random Forest algorithm showed the best accuracy in diagnosing diabetic foot, ahead of neural networks and Naive Bayes with the worst performance.
12.	Ferreira ACBH, Ferreira DD, Oliveira HC, Resende ICD, Anjos A, Lopes MHBDM (2020)/Brazil/Computers in Biology and Medicine^(24)^	Identifying patients with DM who are at high risk of developing diabetic foot, using an unsupervised machine learning technique.	Machine learning/Competitive neural layer-based method^(24)^	Predicting risk and diagnosing diabetic foot ulceration through sociodemographic and clinical variables.	The method was 90% accurate, 70% sensitive, and 100% specific. The use of the method can optimize nursing work by facilitating screening.
13.	Husers J, Hafer G, Heggemann J, Stefan W, Prysucha M, Dissemond J, Mooelleken M, Erfurt-Berge C, Hubner U (2022)/Germany/Studies in health technology and informatics^(25)^	Training an artificial intelligence system and evaluating its performance in diabetic foot detection.	Machine learning/Convolutional neural networks^(25)^	Early identification of patients at risk of diabetic foot ulcer and, consequently, amputation.	The model training showed convergence, with no overfitting. The final model yielded a score of 0.71 on the 108 validation images, with sensitivity of 0.69 and accuracy of 0.67, demonstrating satisfactory validity for classifying images of macerations for clinical use in wound documentation.
14.	Husers J, Hafer G, Heggemann J, Wiemeyer S, John SM, Hubner U (2022)/Germany/Studies in health technology and informatics^(26)^	Developing a stratification scheme that allows for the classification of patients with and without risk of major amputation.	Machine learning/Bayesian method^(26)^	Predicting the risk of amputations in patients with diabetic foot, based on sociodemographic and clinical characteristics.	The system revealed an adequate cut-off point for the risk of amputation of 0.28. Sensitivity and specificity were 0.83 and 0.66. Although the specificity is low, the decision method includes the majority of real patients at risk.
15.	Jayashree J, Vijayashree J (2017)/India/International Journal of Civil Engineering and Technology^(27)^	Proposing a system for predicting the severity of diabetic foot problems using fuzzy expert systems.	Machine learning/Decision manager supported by fuzzy inference^(27)^	Describing the severity of the diabetic foot.	A model is proposed to describe the severity of diabetic foot based on fuzzy expert systems and Wagner’s classification.
16.	Medeiros RA (2015)/Brazil^(28)^	Developing an intelligent diabetic foot prevention monitoring system.	Machine learning^(28)^	Diabetic foot monitoring and self-care.	SIM2PeD consists of a platform integrated with a mobile device to capture data from individuals for monitoring by the medical team and alerts regarding care. Once captured, the information is passed on to the expert system, which generates recommendations based on the care provided. The experiments carried out in a real environment revealed satisfactory and adequate performance for remote monitoring of foot self-care activities.
17.	Nair HKR, Kaur G (2021)/Malaysia/Wounds International^(29)^	Describing the experience of using the TIME tool with diabetic foot patients.	Guiding flowchart^(29)^	Deciding on the treatment plan (wound bed preparation, dressing selection, and management).	Clinical decision support tool based on wound bed preparation, with a view to deciding on the treatment plan according to etiology. The tool facilitated decision-making, guidance, and unification on the appropriate treatment, allowing a systematic approach and communication between professionals.
18.	Nguyen G, Agu E, Tulu B, Strong D, Mombini H, Pedersen P, et al. (2020)/USA/Smart Health^(30)^	Exploring machine learning classifiers to generate actionable decisions in wound care.	Machine learning/Gradient Boosted Machine/Support Vector Machine^(30)^	Deciding on a diabetic foot treatment plan using images.	The Gradient Boosted Machine outperformed other decision algorithms, achieving 81% accuracy, using visual and textual resources. The decisions were (1) continue treatment, (2) request a change in treatment, and (3) refer for specialized treatment.
19.	Peleg M, Shachak A, Wang D, Karnieli E (2009)/Israel/International Journal of Medical Informatics^(31)^	Developing a prototype decision support system based on guidelines to assist in the management of the diabetic foot.	Guiding flowchart^(31)^	Predicting the risk, diagnosing, and treating diabetic foot ulceration, based on guidelines.	Users had a positive response to the prototype, in terms of clarity of design, interaction, and ease of use. The sample expressed a clear intention to use the system in the future, to help with treatment, referrals, risk stratification, and follow-up.
20.	Peng B, Min R, Liao Y, Yu A (2021)/China/Journal of Diabetes Research^(32)^	Determining the accuracy of the new model in predicting the risk of lower limb amputations in the diabetic foot	Guiding flowchart^(32)^	Predicting the risk of diabetic foot amputation through clinical variables.	After identifying the main predictive factors of diabetic foot, a logistic regression was carried out to track the independent factors of amputation, which were applied to build a prediction model. The area under the curve was 0.876 and the calibration curve corrected for the normogram showed a good fit for predicting the risk of amputation. The decision analysis curve indicated that the model was most practical and accurate when the risk threshold was between 6% and 91%.
21.	Schafer Z, Mathisen A, Svendsen K, Engberg S, Thomsen RT, Kirketerp-Moler K (2021)/Denmark/Frontiers in Medicine^(33)^	Understanding the risk factors for diabetic foot and amputation among patients with diabetes, using data from national health registries and machine learning.	Machine learning^(33)^	Predicting the risk of diabetic foot ulceration and amputation through sociodemographic and clinical variables.	The risk of ulceration and amputation is increased in patients with diabetes and cardiovascular complications, peripheral arterial disease, neuropathy, and chronic renal complications. Machine learning proved useful for assessing risk factors for ulceration and amputation, based on secondary data.
22.	Schoen DE, Glance DG, Thompson SC (2015)/Australia/Journal of Foot and Ankle Research^(3)^	Understanding opinions and experiences during the development and evaluation of an electronic diabetic foot risk stratification tool, based on guidelines.	Machine learning/*Software* ^(3)^	Predicting the risk of ulceration based on clinical variables.	The risk tool integrates a simple assessment readily available in a clinical setting and reflects current Australian guidelines, targeting foot examination and investigation of predictors such as previous amputation/ulceration, deformity, presence of pulses, and peripheral neuropathy.
23.	Wijesinghe I, Gamage C, Perera I, Chitranjan C (2019)/Sri Lanka/IEEE Xplore^(34)^	Proposing a prototype of an autonomous system to guide the diagnosis and treatment of diabetic feet.	Machine learning^(34)^	Predicting the risk and diagnosing diabetic foot ulceration.	The system consists of knowledge-based modules for classification based on severity level, clinical decision support and near real-time foot ulcer detection and triage. The average usability score was 88.5, proving to be good but not exceptional.

Regarding the journals and their topics of interest, we found journals related to the development of technologies and innovations in health (n = 7; 30.4%), medicine (n = 5; 21.7%), diabetes and the development of technologies aimed at the disease (n = 4; 17.3%), computer science and engineering (n = 2; 8.6%), and the repository of scientific events in technology (n = 4; 17.3%). One study remained, corresponding to the gray literature.

Eighteen studies (78.2%) were observational studies, nine of which were descriptive (39.1%), three cohort studies (13.0%), two case-control studies (8.6%), two cross-sectional studies (8.6%), one single-case study (4.3%), and one multiple-case study (4.3%). The remainder (n = 5; 21.7%) were methodological studies, concerning the construction of the DSS and its application.

In the field of diabetic foot, expert systems are involved in screening^(9)^, predicting the risk of ulcers and amputations^(3,5,7,15,16,18,21–24,30–34)^, diagnosis^(21,23,24,31,34)^, classifying the stage of severity^(19,25,34)^, deciding on the treatment plan and evaluating the effectiveness of the interventions implemented^(27,28,33)^. One study compared their cost-effectiveness^(20)^.

Various resources were used to train the algorithms. These include processing data relating to the image of the lesion^(7,21,25,30)^, thermal analysis^(15,18)^, plantar grip measurements^(22)^, a questionnaire with clinical and sociodemographic data^(7,16,23–25,32–34)^, the Wagner scale^(7,19)^ and other clinical prediction rules^(15,35)^, alerts relating to remote self-care monitoring^(28)^, and financial analysis between different treatments and amputation^(20)^.

In this context, the main factors related to ulceration, including amputation, were peripheral arterial disease, neuropathy, poor diabetes control, dyslipidemia, cardiovascular disease, chronic kidney complications, and a history of previous ulceration, deformities, inability to feel a 10g monofilament, at least one absent pedal pulse, plantar grip areas, unfavorable family history, smoking and alcoholism, barefoot walking habits, and lack of guidance/care from health professionals regarding the complication^(5,22,33,35)^.

Most expert systems make predictions using supervised or unsupervised machine learning techniques^(9,18,21,28,33,34)^. Among the algorithms used are convolutional neural networks^(19,21)^ for image processing and other decision algorithms^(7,20,24,27,30)^, such as fuzzy inference mechanisms, Gradient Boosted Machine, random forest, support vector machine, multi-layer perception neural network, Naive Bayes, artificial neural networks, competitive neural layer-based method and Radial Base Function.

The introduction of DSS in diabetic foot care was statistically significantly associated with the use of guideline-based practices, improved spending on treatments and interventions, risk factor management, screening, and preventive strategies. In addition, they have reminder, alert, and suggestion resources which, as well as promoting self-care and autonomy, encourage the discussion of therapeutic options, qualifying the professional-patient dialogue in an individualized and unique way^(35–37)^.

However, the main limitations pointed out are due to the insufficient number of studies with the target population, i.e. patients prone to ulceration and amputation, and/or testing limited to community settings with a restricted sample^(9,15,24,25,31,33)^, coupled with the resistance of health professionals to incorporating the tool into routine care^(15,16,18–28,31–34)^, and the incompleteness of the information available in databases^(5,15,18,19,32,33)^.

## DISCUSSION

Mapping the evidence available in the literature reveals that DSS are becoming increasingly relevant in the management and clinical follow-up of DM, including the prevention and care of the diabetic foot. These systems involve supporting health professionals and patients in solving clinical problems by incorporating data from qualitative and quantitative sources, entered manually or automatically into an electronic record system, combined with the experience of specialists and guidelines. Their main purposes include guiding, Qualifying, and unifying the decision-making process^(3,7,30,33)^.

In general, studies on the subject show promising prospects for the incorporation of DSS into the routine of health professionals, as they favor and direct screening, diagnosis, prediction, treatment, risk stratification, referrals, and evaluation of the implementation of the care plan, with an individual and targeted approach, based on patients’ data.

In line with this, DSS have acted to reduce the barriers involved in health care, such as the rational use of resources, integration and transfer of information between primary, specialized, and tertiary care, “clinical inertia” (failure to initiate or intensify therapy when indicated), lack of familiarity with guidelines, protocols, and qualified electronic records, while offering summary reports on patient care, feedback on quality indicators, and benchmarking^(36,38)^.

Similarly, the literature discusses the functionalities of DSS, which mostly include personalized reminders, targeting for risk factor screening, preventive care and clinical tests, assessments for at-risk populations based on history, evidence-based treatment recommendations, including intensification of existing treatment regimens, recommendations for behavioral changes, and alerts for signs of serious risk^(36)^.

There is evidence that DSS with alert, reminder or feedback functionalities are more likely to have an impact on health care. In a randomized clinical trial, the odds ratio of the intervention group versus the control group for the probability of no worsening and improvement was 1.09 (95% CI 0.73; 1.63)^(39)^. Furthermore, a systematic review with meta-analysis confirms that 82% of the DSS available in the literature inferred a significant impact on the care process and, of these, 31% found tangible results with regard to the management of variables associated with DM^(40)^.

Regarding the quality of practices and clinical results related to morbidity and mortality from other conditions (e.g. cancer screening, immunization, CVD prevention), the analysis of randomized clinical trials similarly points to a significant improvement in variables related to screening, requesting clinical exams, and prescribing treatments^(36)^. However, there is scant evidence of its effectiveness in clinical outcomes^(41)^.

In the hospital field, there have been good results in increasing adherence to surgical safety guidelines and protocols, especially with regard to the prescription of perioperative antibiotics, with a reduction in the rate of infections (<1%), qualification of the blood transfusion process and prophylaxis of deep vein thrombosis, inferring savings of more than US$1.6 million annually in a single hospital^(41)^.

There is growing evidence that well-designed and carefully implemented DSS in DM follow-up improve not only the ordering of tests and preventive care, but also enable a dynamic, standardized, and personalized care plan to be drawn up and easily accessed by any member of the healthcare team at any time^(42)^.

Studies on the implementation of DSS in DM, in primary and specialized care, show positive impacts on the control of glycemic index, glycated hemoglobin, blood pressure, and blood cholesterol levels. The systems are generally compatible with the routine practice of institutions and can be integrated with other strategies, such as home visits, educational interventions, case management, and the use of social media^(39,42)^.

For example, a study to build a DSS with a decision manager supported by fuzzy inference for diabetic retinopathy showed an accuracy of 80.76%, sensitivity of 80.67%, and specificity of 85.96%, enabling screening for the complication every three years^(43)^. In the field of clinical decision support for diabetic foot, researchers encourage the promotion of research based on the findings of promoting healing, reducing the risk of death and amputation^(44)^, as well as reflecting the guidelines and protocols for managing the complication^(3)^ and the possibility of reducing errors in diagnosis, risk stratification and functional limitations^(45)^.

The variables most commonly included in the DSS are diabetic neuropathy, peripheral arterial disease, foot deformity, and previous foot complications. These, in turn, are consistently associated with the occurrence of ulceration. The sensitivity of the classifications ranged from 38% to 100%, specificity from 30% to 88%, whereas negative predictive values were always higher than 80% and positive predictive values were always lower than 60%^(44)^. Therefore, the literature shows that health professionals considered the diabetic foot DSS to be easy to use (99%), and believed that it provided useful information for patient care (100%)^(43)^.

As a result, machine learning artificial intelligence methods are increasingly being used in clinical predictive modeling, while modern machine learning approaches such as artificial neural networks and deep learning generally perform better when compared to more traditional methods such as logistic and linear regression^(45)^.

Machine learning mechanisms such as support vector machines, gradient boosted machines, artificial neural networks, random forests, and multi-layer perception neural networks stand out for their satisfactory results, with high accuracy and sensitivity, when it comes to understanding patterns in medical images for predicting, diagnosing, and stratifying diabetic foot disease. However, they require a prior stage of resource extraction and data availability^(18,21,23,34)^. On the other hand, deep learning and Naive Bayes techniques have shown limited accuracy and sensitivity results. However, these methods manage to include the vast majority of patients at real risk of ulceration and amputation^(18,24)^.

Regarding the assessment of the severity of ulcers already installed and the cost of care, the decision manager supported by fuzzy inference showed favorable results, starting with the collection of images and risk factors, followed by the implementation of the Wagner scale and modeling calculations, for data interpretation, support for decision-making regarding treatment, and the generation of alerts^(7)^. In addition to the diabetic foot context, the fuzzy method has shown high sensitivity in other clinical conditions^(46,47)^, around 98%, given that risk prediction considers a set of carefully chosen rules based on the patient’s characteristics. In this respect, the fuzzy method is a potential algorithm for identifying early diagnosis, stratifying risk and monitoring the progress of diabetic foot disease.

The synthesis of information showed the use of the support vector machine method combined with other technologies. Furthermore, this tool shows preferable results in the literature over deep learning and convolutional neural networks, inferring accurate diagnoses (above 99%) in a shorter workflow time, especially from imaging results^(48,49)^. However, the performance of the chosen method may vary depending on the resources used and deployment scenarios, emphasizing that technologies used together have the potential to improve system performance^(48)^.

In addition, the use of case-based reasoning algorithms has proved capable of calculating dissimilarity scores to provide a quantitative measure between a new case and cases stored in the case base^(9)^. For example, the experience with SM2PeD, a platform integrated with a mobile device, shows satisfactory results, based on tests with a real sample, regarding the remote monitoring of self-care activities by the medical team, together with the issuing of alerts and recommendations by the expert system^(28)^. In addition, there is insight into the development of intelligent decision support systems with data generated from remote monitoring based on the Internet of Things.

Thus, clinical prediction rules, guiding flowcharts, and prognostic models, together with expert systems and preventive interventions, have proved to be useful, simple, and effective tools, with satisfactory predictive capacity and low implementation costs, with a view to guiding, unifying, and improving the quality of diagnosis, choice of treatment plan and preventive strategies in diabetic foot^(5,15,29,32)^. In addition, a heuristic decision algorithm for evaluating the cost of medical treatments and diabetic foot amputation provided an early and efficient decision-making process, with the choice of cost-effective treatments and adaptability between different healthcare environments^(28)^.

However, some DSS are limited to recording information and providing generic advice, proving to be not very attractive, which promotes distancing between health professionals and users, hindering the process of implementing smart technologies and promoting self-care strategies^(50)^. The panorama thus highlights the still incipient interaction between clinical practice and expert systems, in terms of usability, acceptance, and recognition of the benefits, substantially evidenced in experimental studies^(36)^.

To this end, an understanding of the models applied is essential, with a view to the usability of the systems, the effectiveness of clinical decision support, and the instruction of those involved in the process. The literature points out that five “rights” must be considered for the successful integration of technology: the right information must be presented to the right audience, in the right format, through the right channels, at the right points in the patients’ lives^(42)^.

Furthermore, the use of DSS should be sparing, restricted to the provision of recommendations, without prejudice to the judgment of health professionals, while also paying attention to the reliability of the data, algorithm, and system itself. Therefore, the availability of reliable sources should be considered before and during the project, reducing negative impacts on patients’ health, methodological limitations, and the presence of biases, which are still seen in health DSS^(51)^, and their negative impact on health outcomes^(52)^.

Allied to this, problems such as the lack of standardization of measurement processes and presentation of results, and low quality in the execution of methods stand out, leading to inconsistent conclusions in some studies^(41)^. To this end, we recommend the development of consistent studies for the design and testing of DSS, including validation with specialists and the target population, implemented in the various scenarios of diabetic foot care practice, to establish results on the effectiveness of their use and impact on outcomes such as cure, hospitalization, amputation, and death, as well as promoting easy and flexible access to these technologies, with a view to training and raising awareness among health professionals^(29,38)^.

The study contributes to expanding knowledge in the field of nursing and health about diabetic foot DSS, bringing new and relevant information about the use of this resource for early diagnosis, appropriate treatment, and continuous monitoring. These systems can help nursing professionals screen at-risk patients, enabling early interventions and early diagnosis of lesions. It also helps support clinical decision-making and evidence-based guidelines for treatment, promoting standardized and personalized care.

The limitations of this scoping review include the difficulty in understanding the context in which DSS are applied and their contribution to clinical practice. In addition, the presence of heterogeneous studies makes it difficult to directly compare the studies and synthesize the results.

## CONCLUSION

Decision support systems corroborate the orientation and qualification of clinical practice, with regard to screening, diagnosis, prediction, treatment, risk stratification, and evaluation of the diabetic foot care plan, using artificial intelligence and machine learning resources, which stand out for their satisfactory results, with high precision and sensitivity, inferring excellent prospects for their incorporation into clinical practice.
